# In vitro study on the effect of cornin on the activity of cytochrome P450 enzymes

**DOI:** 10.1186/s12906-021-03309-y

**Published:** 2021-05-09

**Authors:** Qun Zhang, Zengqiang Qu, Yanqing Zhou, Jin Zhou, Junwei Yang, Shengjian Li, Qiuping Xu, Xuedong Zhou

**Affiliations:** 1 Shanghai Baoshan Aged-nursing hospital, Shanghai, 201900 China; 2 Department of Invasive Technology, Shanghai Eastern Hepatobiliary Surgery Hospital, Shanghai, 200438 China; 3 Department of Pharmacy, Shanghai Baoshan Luodian Hospital, No.121 Luoxi Road, Baoshan District, Shanghai, 201908 China; 4 Clinical research center, Shanghai Baoshan Luodian Hospital, No.121 Luoxi Road, Baoshan District, Shanghai, 201908 China

**Keywords:** Cornin, CYP3A4, CYP2C9, CYP2E1, Drug-drug interaction

## Abstract

**Background:**

Cornin is a commonly used herb in cardiology for its cardioprotective effect. The effect of herbs on the activity of cytochrome P450 enzymes (CYP450s) can induce adverse drug-drug interaction even treatment failure. Therefore, it is necessary to investigate the effect of cornin on the activity of CYP450s, which can provide more guidance for the clinical application of cornin.

**Methods:**

Cornin (100 μM) was incubated with eight isoforms of CYP450s, including CYP1A2, 2A6, 3A4, 2C8, 2C9, 2C19, 2D6, and 2E1, in pooled human liver microsomes. The inhibition model and corresponding parameters were also investigated.

**Results:**

Cornin exerted significant inhibitory effect on the activity of CYP3A4, 2C9, and 2E1 in a dose-dependent manner with the IC_50_ values of 9.20, 22.91, and 14.28 μM, respectively (*p* < 0.05). Cornin inhibited the activity of CYP3A4 non-competitively with the *Ki* value of 4.69 μM, while the inhibition of CYP2C9 and 2E1 by cornin was competitive with the *Ki* value of 11.31 and 6.54 μM, respectively. Additionally, the inhibition of CYP3A4 by cornin was found to be time-dependent with the *K*_*I/*_*K*_*inact*_ value of 6.40/0.055 min^− 1^·μM^− 1^.

**Conclusions:**

The inhibitory effect of cornin on the activity of CYP3A4, 2C9, and 2E1 indicated the potential drug-drug interaction between cornin and drugs metabolized by these CYP450s, which needs further investigation and validation.

## Background

With the popularization of Chinese traditional medicine (CTM), CTM has begun to be used worldwide. In CTM, prescriptions are mixtures that contain at least two types of herbs. Herb-herb interactions or herb-drug interactions are important factors that affect the pharmacokinetics and metabolism of drugs and even cause toxicity. Cytochrome P450 enzymes (CYP450s) are a family of heme-containing proteins that play important roles in the phaseI metabolism of most clinical drugs in the liver and intestine. The activities of CYP450s directly affect the biotransformation and metabolism of various drugs. For example, puerarin is widely used in the treatment of cardiovascular diseases and diabetes, and it can inhibit the activity of CYP3A4. Several previous studies demonstrated that the inhibitory effect of puerarin on the activity of CYP3A4 resulted in adverse effects on the pharmacokinetics of various drugs, such as triptolide, edaravone, and astragaloside IV [1–3].

Cornin is a herb that is extracted from the fruit of *Verbena officinalis* L. and is commonly used in cardiology, which can exert cardioprotective effects [4]. Cornin was also reported to significantly decrease blood pressure, reverse cardiac hypertrophy, and improve heart function [5, 6]. Cornin is always co-administrated with other drugs to make the treatment more efficient. Previous studies have mainly focused on the pharmacodynamic effects of cornin, and whether cornin can affect the metabolism of co-administered drugs by regulating metabolic enzymes is still unknown. Therefore, it is urgent to investigate the interaction between cornin and CYP450s and determine the effect of cornin on the activities of CYP450s, such studies could provide guidance for improving clinical application of herbs.

CYP1A2, 2A6, 3A4, 2C8, 2C9, 2C19, 2D6, and 2E1 are major CYP450 isoforms, that are responsible for the metabolism of most clinical drugs. This study investigated the effects of cornin on the activities of these CYP450 isoforms in pooled human liver microsomes (HLMs) with the employment of the enzyme kinetic study that included probe substrates and reactions.

## Methods

### Chemicals and reagents

**Fig. 1:**
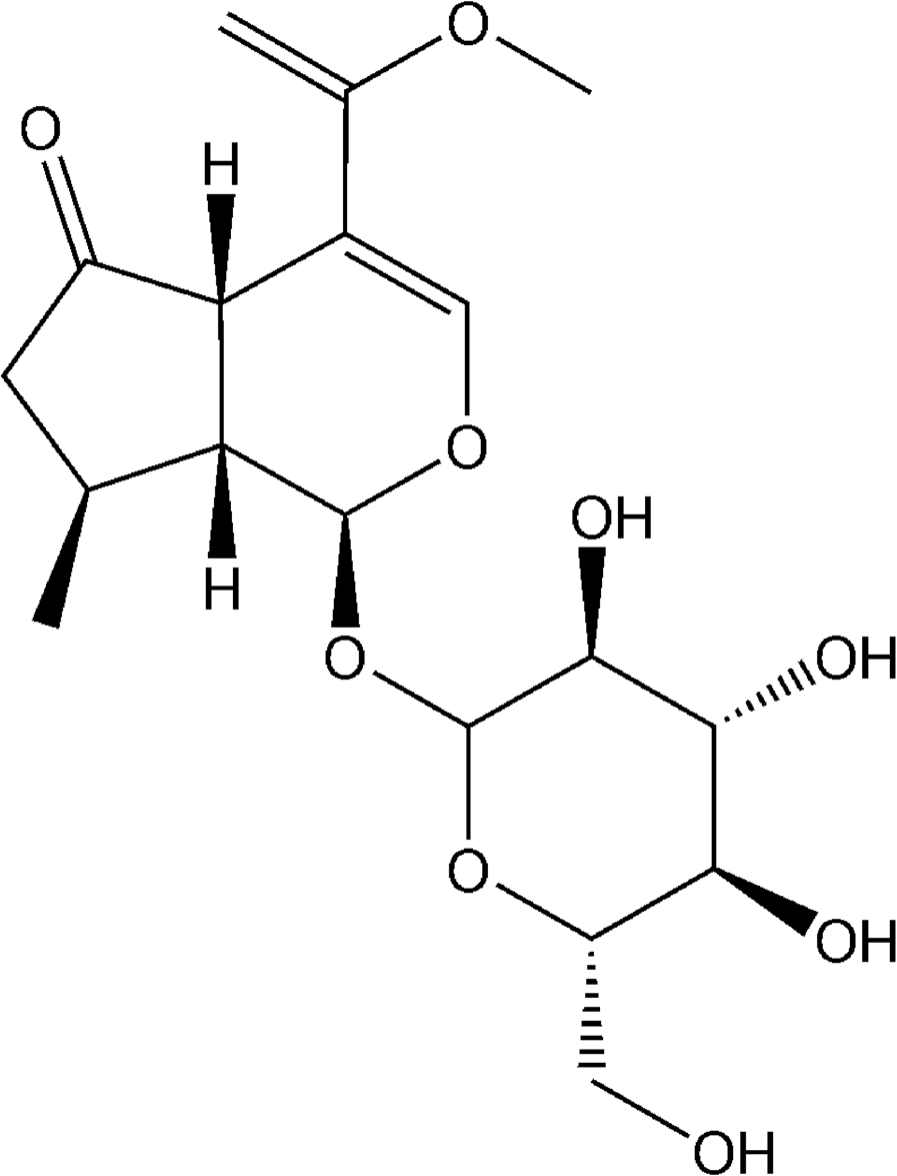
The chemical structure of cornin

Cornin (≥ 98%, Fig. 1) and testosterone (≥ 98%) were purchased from the National Institute for the Control of Pharmaceutical and Biological Products (Beijing, China). D-glucose-6-phosphate, glucose-6-phosphate dehydrogenase, 4′-hydroxydiclofenac (≥ 98%), 4-hydroxymephenytoin (≥ 98%),NADP+, phenacetin (≥ 98%), acetaminophen (≥ 98%), 6β-hydroxytestosterone (≥ 98%), corticosterone (≥ 98%), 7-hydroxycoumarin (≥ 98%), sulfaphenazole (≥ 98%), tranylcypromine (≥ 98%), chlorzoxazone (≥ 98%), quinidine (≥ 98%), 6-hydroxychlorzoxazone (≥ 98%), paclitaxel (≥ 98%), clomethiazole (≥ 98%), and furafylline (≥ 98%) were obtained from Sigma Chemical Co. Montelukast (≥ 98%) was obtained from Beijing Aleznova Pharmaceutical (Beijing, China). Coumarin (≥ 98%), dextromethorphan (≥ 98%), diclofenac (≥ 98%), and ketoconazole (≥ 98%) were purchased from ICN Biomedicals. Pooled HLMs were purchased from BD Biosciences Discovery Labware. All other reagents and solvents were of analytical reagent grade.

### Assay with human liver microsomes

An incubation system constituted of 100 mM potassium phosphate buffer (pH 7.4), the appropriate concentration of HLMs, a corresponding probe substrate and cornin (or positive inhibitor for different probe reactions) and an NADPH generating system, including 1 mM NADP^+^, 10 mM glucose-6-phosphate, 1 U/mL of glucose-6-phosphate dehydrogenase, and 4 mM MgCl_2_ was performed with the final volume of 200 μL. The final microsomal protein concentration, incubation time for specific probe reactions and concentration of substrates are summarized in Table 1, according to previous studies [7–9]: phenacetin O-deethylation for CYP1A2, coumarin 7-hydroxylation for CYP2A6, testosterone 6β-hydroxylation for CYP3A4, paclitaxel 6α-hydroxylation for CYP2C8, diclofenac 4′-hydroxylation for CYP2C9, S-mephenytoin 4-hydroxylation for CYP2C19, chlorzoxazone dextromethorphan O-demethylation for CYP2D6, and 6-hydroxylation for CYP2E1. All incubations were performed in triplicate, all data were presented as mean value ± SD.

**Table 1:** Isoforms tested, marker reactions, incubation conditions, and K_m_ used in the inhibition study. The reaction conditions were performed according to previous studies [7–10]

CYPs	Marker reactions	Substrate concentration (μM)	Protein concentration (mg/mL)	Incubation time (min)	Estimated K_m_ (μM)	Inhibitor concentration (μM)	Inhibitors
1A2	phenacetin O-deethylation	40	0.2	30	48	10	furafylline
3A4	testosterone 6β-hydroxylation	50	0.5	10	53	1	ketoconazole
2A6	coumarin 7-hydroxylation	1.0	0.1	10	1.5	10	tranylcypromine
2E1	chlorzoxazone 6-hydroxylation	120	0.4	30	126	50	clomethiazole
2D6	dextromethorphan O-demethylation	25	0.25	20	4.8	10	quinidine
2C9	diclofenac 4′-hydroxylation	10	0.3	10	13	10	sulphaphenazole
2C19	S-Mephenytoin 4-hydroxylation	100	0.2	40	105	50	tranylcypromine
2C8	paclitaxel 6α-hydroxylation	10	0.5	30	16	5	montelukast

Except for dextromethorphan and quinidine dissolved in water, the other probe substrates, positive inhibitors and cornin were dissolved in 1% (v/v) methanol. The concentration of cornin was 100 μM and the positive inhibitor concentrations were summarized in Table 1.

After a pre-incubation of 3 min at 37 °C, an NADPH-generating system was added to initiate the reaction. The reaction was terminated by the addition of 100 μL acetonitrile (10% trichloroacetic acid for CYP2A6), and the solution was placed on ice. After centrifuging at 12,000 rpm for 10 min, the supernatant was analyzed by the HPLC analysis with the employment of Agilent 1260 series instrument with DAD and FLD detector, and the quantitative assay for the corresponding metabolites was performed as previously reported [9, 10].

### Enzyme inhibition and kinetic studies of cornin

An enzyme inhibition study was performed to investigate the effect of cornin on the activity of eight CYP isoform. Then, CYPs of which the activity was affected by cornin were chosen for the kinetic study with 0–50 μM cornin and different concentration of probe substrates (20–100 μM testosterone for CYP3A4, 2–20 μM diclofenac for CYP2C9 and 25–250 μM chlorzoxazone for CYP2E1) to obtain the corresponding parameters, including concentration (IC_50_) and *Ki* values.

### Time-dependent inhibition study of cornin

The HLMs (1 mg/mL) was pre-incubated with 20 μM cornin with an NADPH-generating system for 30 min at 37 °C. After the pre-incubation, 20 μL aliquot was incubated with an NADPH-generating system and probe substrates with specific concentrations (approximate to Km) in another incubation tube to evaluate the residual activity. The corresponding metabolites were analyzed at 0, 5, 10, 15, and 30 min of the incubation by HPLC.

A high probe substrate concentration (approximately 4-fold Km values) and various concentrations of cornin (0–50 μM) were used to obtain the values of the inactivation constant (*KI*) and the rate of inactivation (*K*_*inact*_) after different preincubation times (0–30 min), with a two-step incubation scheme, as described above.

### Statistical analysis

The least-squares linear regression of the inverse substrate concentration versus the inverse velocity (Lineweaver-Burk plots) was used to obtain the enzyme kinetic parameters, and the mean values were used to calculate Vmax and Km. Inhibition data from the experiments that were conducted using multiple compound concentrations were represented by Dixon plots, and inhibition constant (*Ki*) values were calculated using non-linear regression according to the following equation:

$$ \mathrm{v}=\left(\mathrm{VmaxS}\right)/\left(\mathrm{Km}\left(1+\mathrm{I}/ Ki\right)+\mathrm{S}\right), $$

where I is the concentration of the compound, *Ki* is the inhibition constant, S is the concentration of the substrate, and Km is the substrate concentration at half the maximum velocity (Vmax) of the reaction. The mechanism of the inhibition was inspected using the Lineweaver-Burk plots and the enzyme inhibition models. The data comparison was performed using Student’s t-test and performed using IBM SPSS statistics 20 (SPSS Inc.).

## Results

### Effects of cornin on the activities of CYP450s

**Fig. 2:**
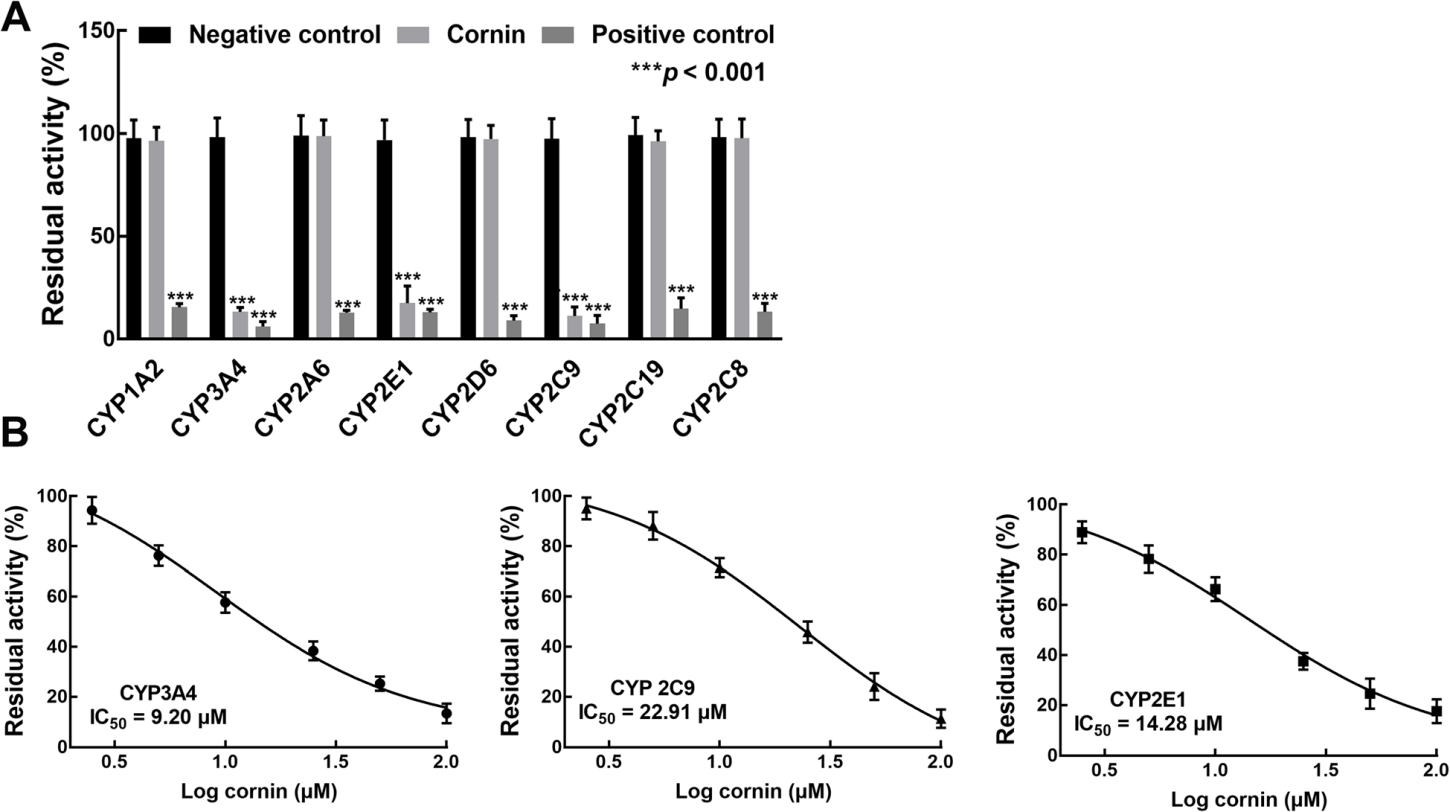
Effect of cornin on the activity of CYP450s. **a** Cornin significantly inhibited the activity of CYP3A4, 2C9, and 2E1. *p < 0.001 relative to negative controls. **b** The dose-dependent experiments of CYP3A4, 2C9, and 2E1

As shown in Fig. 2a, the activities of CYP3A4, 2C9, and 2E1 were significantly inhibited by cornin compared with the blank control (*p* < 0.05), while other CYP isoforms were not affected by cornin (*p* > 0.001). The residual activities of CYP3A4, 2C9, and 2E1 in pooled HLMs were decreased to 13.35, 11.33, and 17.62% after incubating with 100 μM cornin. The inhibitory effect of cornin was lower than that of specific inhibitors and the difference was not significant (*p* > 0.05).

Then, CYP3A4, 2C9, and 2E1 were incubated with 0–100 μM cornin, and the inhibition of these CYP450s was found to occur in a dose-dependent manner with IC_50_ values of 9.20, 22.91, and 14.28 μΜ, respectively (Fig. 2b).

### Model of CYP3A4, 2C9, and 2E1 inhibition by cornin

**Fig. 3:**
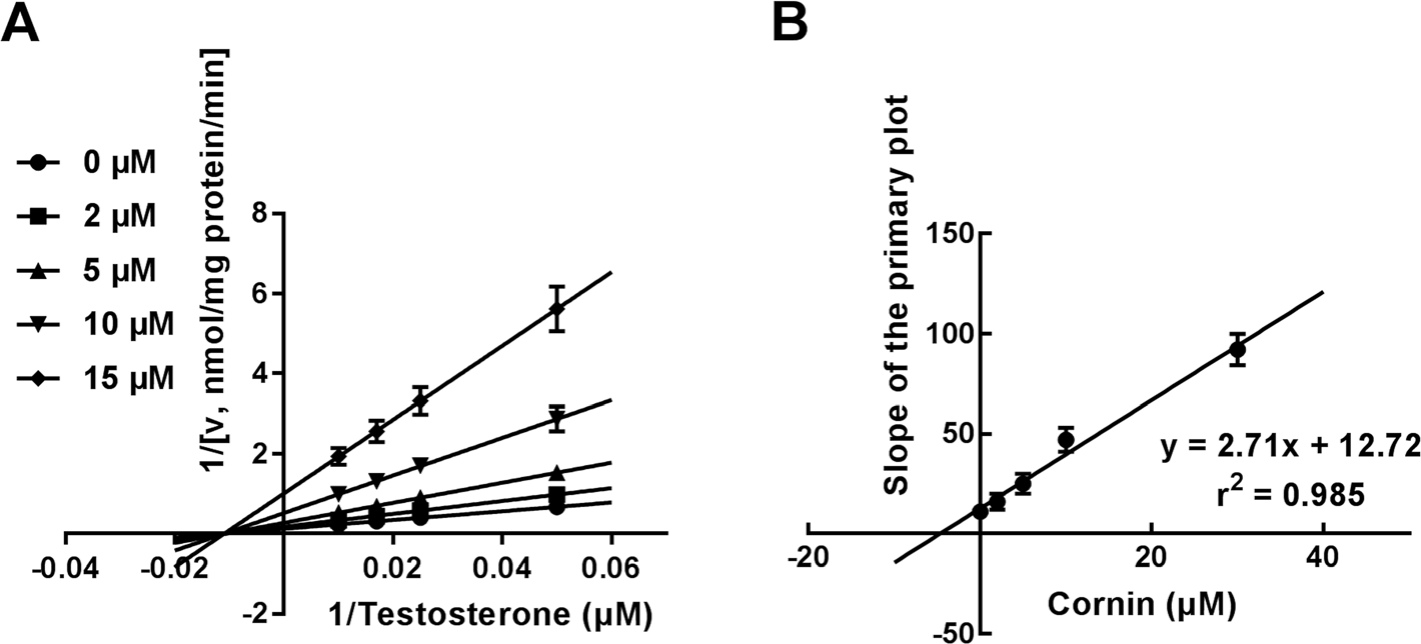
The inhibition of CYP3A4 by cornin. **a** Lineweaver-Burk plots of the inhibition of CYP3A4 by cornin in the presence of 20–100 μM testosterone and 0–15 μM cornin. The inhibition of CYP3A4 by cornin was performed non-competitively. **b** The secondary plot for *Ki* of the inhibition of cornin on CYP3A4

**Fig. 4:**
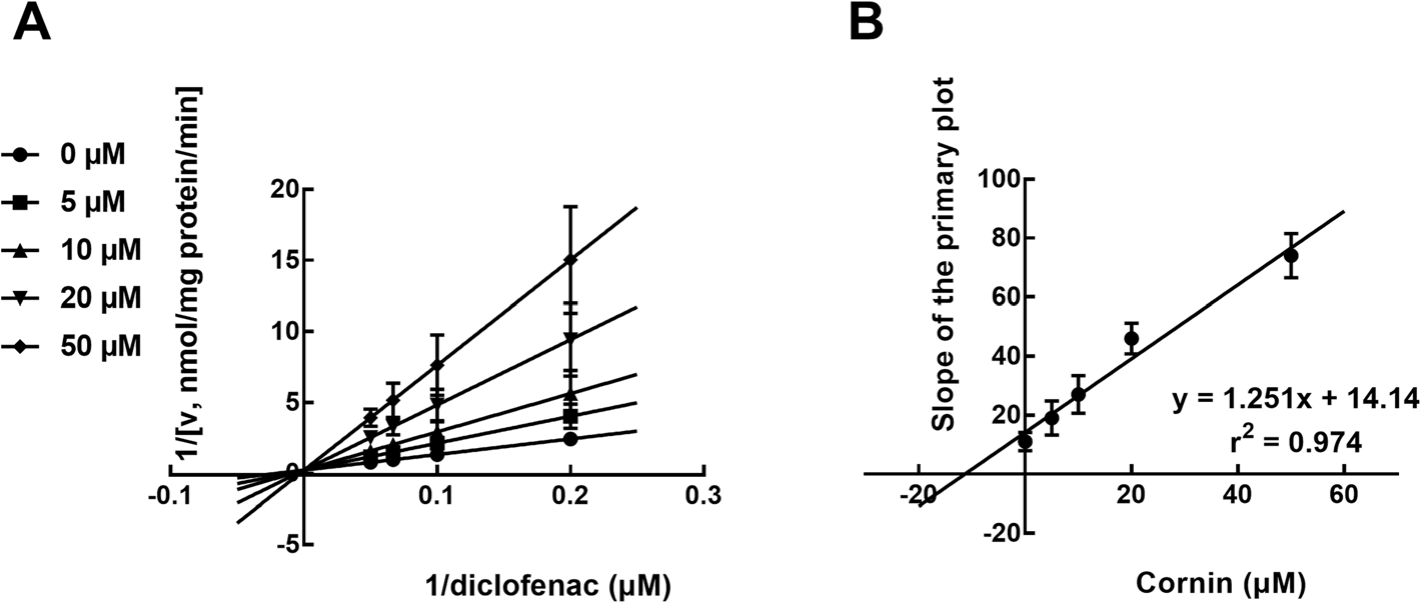
The inhibition of CYP2C9 by cornin. **a** Lineweaver-Burk plots of the inhibition of CYP2C9 by cornin in the presence of 2–20 μM diclofenac and 0–50 μM cornin. The inhibition of CYP2C9 by cornin was performed competitively. **b** The secondary plot for *Ki* of the inhibition of cornin on CYP2C9

**Fig. 5:**
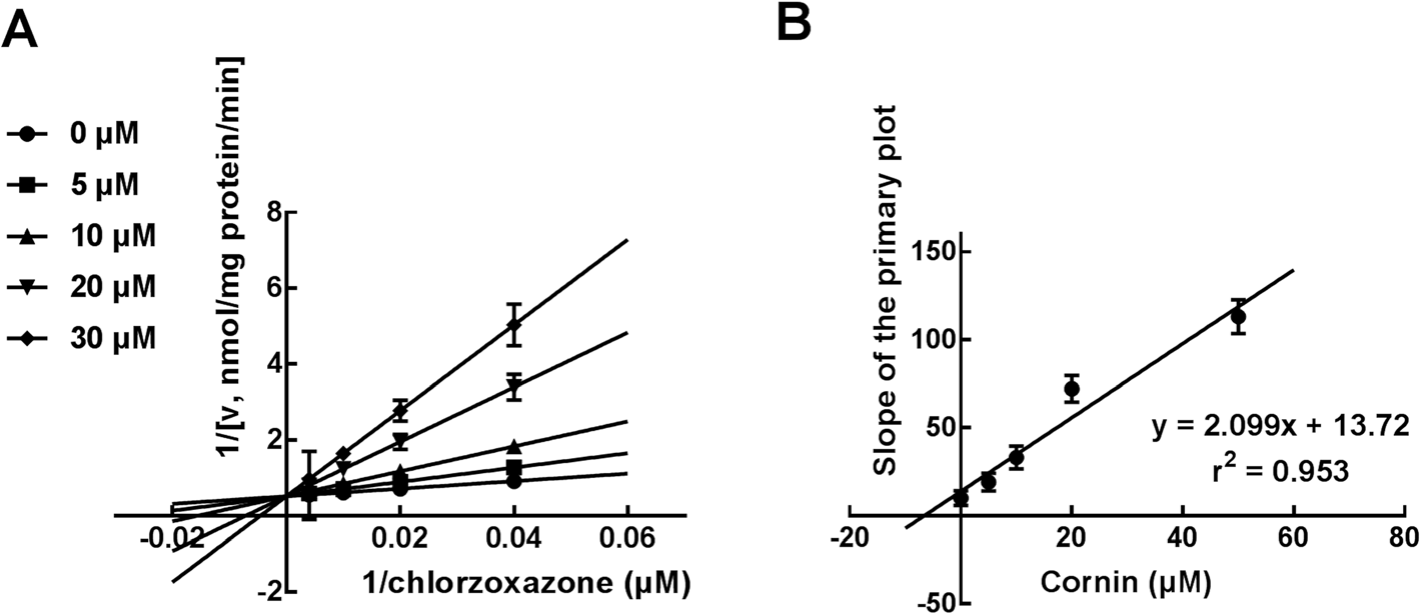
The inhibition of CYP2E1 by cornin. **a** Lineweaver-Burk plots of the inhibition of CYP2E1 by cornin in the presence of 25–250 μM chlorzoxazone and 0–30 μM cornin. The inhibition of CYP2E1 by cornin was performed competitively. **b** The secondary plot for *Ki* (B) of the inhibition of cornin on CYP2E1

With the help of Lineweaver-Burk plots of inhibitory kinetic data, the inhibition of CYP3A4 by cornin was best fitted in a noncompetitive manner (Fig. 3a). Moreover, the *Ki* value was determined to be 4.69 μM according to the secondary Lineweaver-Burk plot (Fig. 3b). With the employment of the Lineweaver-Burk plots, the inhibition of CYP2C9 was found to be competitive (Fig. 4a), while further secondary fitting analysis obtained *Ki* value of CYP2C9 as 11.31 μM (Fig. 4b). Similarly, the Lineweaver-Burk results showed that cornin inhibited CYP2E1 in a competitive manner (Fig. 5a), and the *Ki* value was 6.54 μM (Fig. 5b).

### Time-dependent inhibition of CYP3A4 by cornin

**Fig. 6:**
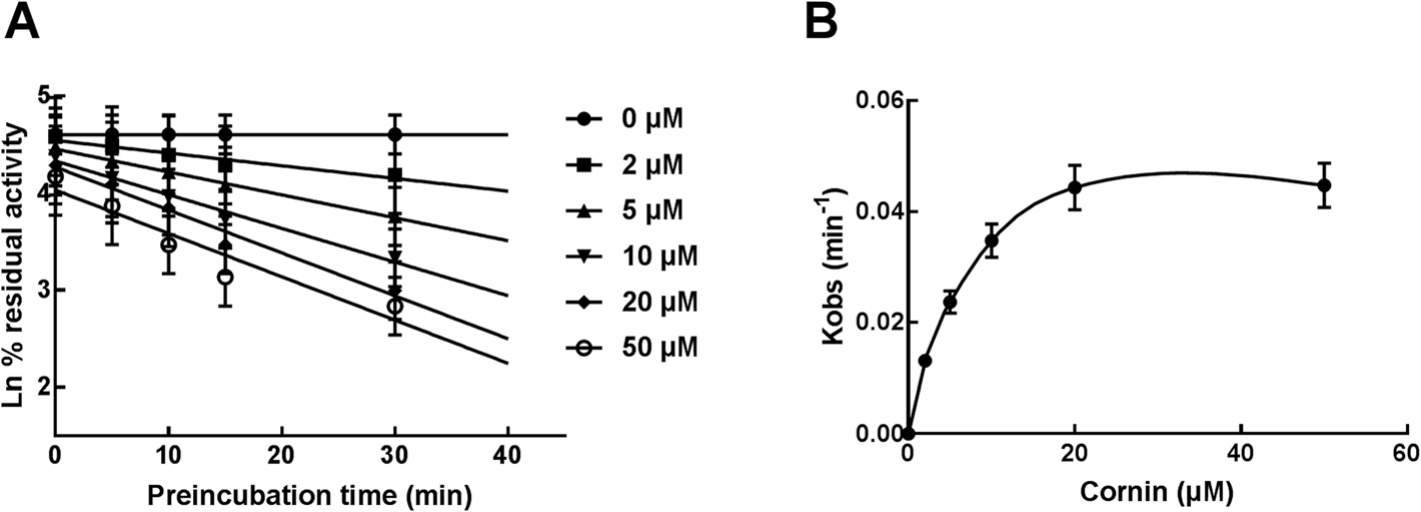
Effect of incubation time on the inhibition of CYP3A4 by cornin and corresponding parameters. **a** The initial rate constant of inactivation of CYP3A4 by each concentration (*K*_*obs*_) was determined through linear regression analysis of the natural logarithm of the percentage of remaining activity versus pre-incubation time. **b** The *K*_*I*_ and *K*_*inact*_ values were determined through non-linear analysis of the *K*_*obs*_ versus the cornin concentration

CYP3A4, 2C9, and 2E1 were incubated with 20 μM cornin for 0–30 min to investigate the effect of incubation time on the inhibitory effect of cornin. The activity of CYP3A4 was significantly decreased with incubation time, but CYP2C9 and 2E1 were not affected (data not shown). Then, CYP3A4 was incubated with 0–50 μM cornin for 0–30 min to characterize the time-dependent inhibition of CYP3A4 by cornin (Fig. 6a). With the help of nonlinear regression analysis, the value of *KI/K*_*inact*_ was calculated to be 6.40/0.055 min^− 1^ μM^− 1^, indicating that 5.5% CYP3A4 was further inactivated every minute in the presence of 6.4 μM cornin (Fig. 6b).

## Discussion

Multiple-drug therapy could provide simultaneous treatment for concurrent diseases and complex chronic disorders, which leads to its wide application in the clinic [11, 12]. The co-administration of various drugs leads to adverse reactions, such as toxicity and treatment failure. The inhibition or induction of CYP450 activity plays vital roles in the metabolism and pharmacokinetics of various drugs during drug-drug interactions [13]. For example, the co-administration of nobiletin and glycyrrhizin induced adverse interactions, which resulted in a decrease of in the plasma concentration of nobiletin due to the enhancement of CYP3A4 activity by glycyrrhizin [14]. Cornin is one of the most widely used herbs in cardiology and exerts the effects of decreasing blood pressure, reversing cardiac hypertrophy, and improving heart function [5, 6]. It is essential to investigate the effects of cornin on the activities of CYP450s.

Here, cornin was incubated with eight major isoforms of CYP450s in pooled HLMs to determine the effect of cornin on these CYP450s. It was found that cornin significantly inhibited the activities of CYP3A4, 2C9, and 2E1, which are responsible for the metabolism of the vast majority of drugs [13]. CYP3A4 is one of the most abundant CYP isoforms in the human liver and intestine and are involved in the metabolism of a large number of drugs [15, 16]. The vital functional role of CYP3A4 has been widely shown, and various drug-drug interactions mediated by CYP3A4 have also been reported. The inhibition of CYP3A4 by cornin implied a potential interaction between cornin and drugs metabolized by CYP3A4. Additionally, the inhibition of CYP3A4 was achieved in a dose-dependent manner and time-dependent manner. The IC_50_ value of CYP3A4 is a vital factor for evaluating the toxicity and clinical risk associated with cornin and provides guidance for dose of cornin used in the clinic. In the clinic, care is warranted in the dosing of CYP3A4 substrates that are co-administrated with cornin, and dose may be adjusted with incubation time.

In addition, cornin was also found to competitively inhibit the activities of CYP2C9 and 2E1, which may be a result of the similar structures of cornin and CYP2C9 and 2E1 substrates, such as hydroxyl and aromatic functional groups. CYP2C9 and 2E1 are involved in the metabolism of a large number of drugs, thuscontributing to the wide variability in pharmacokinetics in the metabolism of drugs [17, 18]. For example, the metabolism of warfarin was inhibited by cannabis due to the inhibition of CYP2C9 by cannabis [19]. *Kaempferia parviflora* extract accelerates the metabolism of acetaminophen by inducing the activity of CYP2E1 [20]. Therefore, the obtained findings also suggested a potential risk associated with the co-administration of cornin and drugs metabolized by CYP2C9 and 2E1.

Currently, the application of in silico analysis for mechanically assessing the interaction between different drugs and CYP450s has drawn special attention, and this approach provides future prospects for further studies [21–24]. Furthermore, this in vitro study revealed the inhibitory effect of cornin on the activities of CYP3A4, 2C9, and 2E1. The lack of in vivo pharmacokinetic data on cornin is another limitation of this study, and such data could help assess the clinical significance of the obtained IC_50_ values. The in vivo interaction and potential drug-drug interaction need to be verified by additional in vivo experiments.

## Conclusion

In vitro findings in this study indicated an inhibitory effect of cornin on the activities of CYP3A4, 2C9, and 2E1. Cornin was identified as a noncompetitive inhibitor of CYP3A4 and a competitive inhibitor of CYP2C9 and 2E1. These results suggested the potential drug-drug interaction between cornin and CYP3A4, 2C9, and 2E1 substrates in the clinically co-administrated prescriptions, and these results require further in vivo validation in future investigations.

## Data Availability

The datasets used and/or analysed during the current study are available from the corresponding author on reasonable request.

## Abbreviations

CYP450s: Cytochrome P450 enzymes
CTM: Chinese traditional medicine
HLMs: Human liver microsomes
